# Chicago Gynecological Society

**Published:** 1885-01

**Authors:** 

**Affiliations:** 2330 Indiana avenue


### SOCIETY PROCEEDINGS.

CHICAGO GYNECOLOGICAL SOCIETY.

*Regular Meeting. Dec. 19th, 1884.*—The President, Dr. H. P. Merriman, in the chair.

Dr. W. H. Byford read a paper, entitled "A Case of Mural Pregnancy." The history of the case was obscure. The patient was twenty-eight years old, married seven years, had one child, six years old. She supposed she became pregnant, for the second time, in February, 1883. In April she became fatigued, and had hemorrhages, which continued until May 9th—about four weeks. Oct. 14th, a discharge of yellow fluid, about one gallon in quantity, occurred. A putrescent, sero-sanguineous discharge followed, continuing three months. Jan., 1884, a large, brownish mass, with very fœtid odor, was expelled. After this event, mensturation occurred until July. In May, she was quite large, and had bearing-down pains. She entered the hospital Oct. 6th, 1884. She was tapped Oct. 18th, and about four quarts of thick, tenacious fluid, resembling the fluid of an ovarian cyst, was removed. This fluid coagulated on the addition of nitric acid and on boiling. Assisted by Dr. R. Tilley, a microscopical examination was made, with negative results. The "Drysdale" cell was not found. Laparotomy was performed, and a fœtus with placenta was removed without hemorrhage or difficulty. In order to secure perfect drainage, it was considered best to remove the uterus. The operation was performed on Oct. 30th; the patient did not react, but died within twenty-four hours. Prior to the operation, the patient was extremely reduced by her protracted sufferings. Dr. Byford, in a similar case, at the present time, would elect the vaginal operation. The specimens removed from the woman were exhibited as supporting the diagnosis of mural pregnancy.

This was the second case of mural pregnancy that had come under the reader's observation within a period of five years. The first case was reported to the Chicago Gynecological Society some time ago. The patient was in labor and moribund when Dr. Byford saw her. She had been in labor until exhausted. There was no difficulty in making a diagnosis. The head was low down in the pelvis, almost on the perineum. The *os uteri* was well-nigh inaccessible behind and above the symphysis. The body of the uterus, somewhat enlarged, could be felt, in the lower and anterior part of the abdomen, attached to the tumor containing the fœtus. The fœtus could be felt through the abdominal wall, surrounded by a thick involucrum, apparently as thick as the uterine walls. Fœtal extremities could be distinguished. When dissected, the sac, in which the fœtus was contained, was found to consist of a thick layer of muscular fibers. These fibers were directly continuous with those of the uterus. The tubes and ovaries lay on either side of the lower portion of the sac. The fecundated ovum had made its way down the tube, became lodged in a diverticulum in the anterior wall, and was gradually extruded into the cavity of the abdomen. The fœtus was thus developed within the uterus, though not within the uterine cavity. The resemblance to normal pregnancy is great in the presentation and position of the fœtus, deep down in the pelvic cavity, behind the vagina. The head, in this case, was fixed by the concentric contraction of the uterine fibers, by which it was surrounded, and could be easily outlined as it lay there covered by the posterior vaginal wall.

The specimen presented is much less perfect than the one described, because of the numerous effects wrought upon it during the great length of time it remained in the maternal body, and the mutilation, consequent upon enucleation.

The treatment of these cases ought to be considered apart from that of extra-uterine pregnancy at term. It is always a matter for special consideration, in connection with each case, as it presents itself, whether or no the removal of the fœtus, at term, in extra-uterine gestation, should be attempted. The dangers of laparotomy are greatly increased by the inability to remove the placenta. The surface to which it is attached has no contractile power, so that the divided vessels are left patulous. If hemorrhage does not immediately prove fatal, the blood is a source of sepsis that must almost certainly destroy the patient. Laparotomy would more likely prove successful if performed some days after the death of the child. In these cases of ectopic or interstital uterine pregnancy, the fœtus may be easily removed through the vagina. An incision made through the posterior vaginal wall would completely uncover the presenting part and enable one to apply the forceps or attack it with the perforator and crotchet as in ordinary labor. After the removal of the fœtus, the placenta should be allowed to separate spontaneously.

Since writing this report, Dr. Byford has seen a case reported in the *Annales de Gynecologie*, July, 1884, occurring in the practice of Mr. Matheson, of England, illustrative of the execution of this plan. The case was reported to the London Obstetrical Society under the title, "Extra-uterine Pregnancy, the extraction of a living fœtus through the vagina." The child was slightly asphyxiated, but recovered. A sponge, saturated with perchloride of iron was introduced into the sac, after removal of the placenta. The mother recovered. It would seem that the author did not suspect his case to be one of interstital pregnancy. During the discussion that followed, only one of those present expressed the opinion that it was of that variety. Mr. Griffith thought it was either interstital pregnancy, or one in which the fœtus was developed in one portion of a double uterus.

*Discussion.*—Dr. Edward Warren Sawyer thought that interstitial pregnancy meant the development of the ovum in the uterine portion of one of the tubes. In Dr. Byford's case the uterine portions of the tubes were not involved. It reminded him of a case he had seen near Denver. In this case, a secondary uterus, with muscular walls, had been developed, but as the tubes were not involved, he did not feel justified in designating the case one of interstitial pregnancy.

Dr. D. T. Nelson said, with reference to the treatment of the placenta, that Dr. Byford's advice was that usually recommended in the text-books. The placenta should be left alone in those cases in which the walls of the secondary uterus were not muscular. He had seen a case in the museum of the Chicago Medical College, in which no muscular fibre could be detected in the walls. When the walls of the adventitious uterus were muscular it was questionable whether or no the placenta should be left alone. If the placenta is removed, there is danger of hemorrhage; if the placenta remains, there is danger of sepsis. When there was reason to suppose that contractions of the adventitious uterus would check hemorrhage, he thought the placenta should be removed. He had no experience in these cases.

Dr. E. C. Dudley replied to Dr. Nelson. Women, in cases of extra-uterine pregnancy, in which the placenta has been allowed to remain, do not die of sepsis. He had seen two or three cases in which the sac had been united to the abdominal incision. Whenever evidence of sepsis occurred, the sac was washed out and the temperature immediately fell to the normal. The placenta, under these circumstances, is spontaneously eliminated in about three weeks.

It required phenomenal powers of diagnosis to tell in the concrete case whether or no the sac had sufficient muscular fibers to prevent hemorrhage. The placenta should be permitted to remain within the sac.

Dr. J. H. Etheridge thought that if, on microscopical examination, it was found that the muscular fibers of the normal uterus were continuous with those of the adventitious uterus, the case was one of mural pregnancy. In cases of abdominal pregnancy, there was a line of demarcation between the normal and the adventitious uterus.

Dr. A. Reeves Jackson thought the members of the Society were greatly indebted to Professor Byford for the presentation of such an interesting specimen. He thought, however, with Dr. Sawyer, that the results of the anatomical investigation did not support the author's diagnosis. The uterine portions of the tubes were not involved. So valuable a specimen deserved very close microscopical and macroscopical examinations. It ought to be referred to a competent pathological anatomist.

Dr. John Bartlett thought the ovum had not passed through the tube, but had been developed in the broad ligament, beneath the peritoneum, and had, in this manner, derived muscular fibers from the uterus.

Dr. W. W. Jaggard referred to the fact that next to ovarian pregnancy, interstitial pregnancy was of most infrequent occurrence. Up to the present time, about thirty cases, in regard to which the diagnosis was positive, had been reported. Interstitial or mural pregnancy included other sites of development than the uterine portions of the tubes. Dr. Gilbert's case, reported in the Boston *Medical and Surgical Jonrnal*, 3rd March, 1877, and alluded to by Professor Lusk in his treatise on midwifery, was a case in point. The ovum, in this case, was developed, in what seemed to be a bifurcation of the Fallopian tube. In Dr. Byford's case, the tubo-uterine orifices were not involved. The sac was extrinsic to the uterine walls. It was probably a case of abdominal pregnancy, in which the ovum became attached to the posterior wall, and derived muscular fibers from this locality. The fact, that a continuity of muscular fibers from the normal uterus to the adventitious uterus might be ascertained upon microscopical examination, would prove nothing as to the nature of the pregnancy. Dr. Byford's case resembled that of Janvrin, in which the ovum lodged on the posterior wall of the uterus. The specimen was worthy of more exact investigation and should be placed in the hands of a competent pathological anatomist.

Dr. Sawyer said that abdominal pregnancy, with location of ovum on posterior uterine wall, was not at all improbable. He then referred to Bischoff's and Leopold's observations and experiments with relation to the "external wandering over of the egg." Beigel had ridiculed the idea. It was like a blind man introduced into a large, empty room, with a thread in his hand, seeking to find and thread the eye of a needle located in some indefinite quarter of the room. Notwithstanding this sarcasm, the fact of the external wandering over of the egg was a matter of positive knowledge. The egg may pass from one ovary to the opposite Fallopian tube through the abdominal cavity. He thought the specimen exhibited was one of abdominal pregnancy.

Dr. Dudley thought the fact of the external wandering over of the egg was not disputed at the present time. Playfair, in his "Treatise on Midwifery," gave a clear exposition of the subject.

Dr. Charles Warrington Earle said that the fact of external wandering over of the egg was fully recognized twelve years ago.

Dr. Sawyer said the ovum in abdominal pregnancy might be attached to the posterior wall of the uterus, the mesentery under surface of the liver, or to other viscera.

Dr. Nelson made the remark that in both of the cases cited by Dr. Byford, *deciduæ* had been cast off by the *uteri*.

Dr. Jackson said that Fränkel was of the opinion that the formation and extrusion of a decidua was a constant occurrence in extra-uterine pregnancy. It was pathognomonic of the condition.

Dr. W. H. Byford was not surprised that certain members did not agree with him in his diagnosis. He thought that in the first case the fecundated ovum passed through the tube, but had found some diverticulum in the uterine cavity, and had passed into the uterine wall, had developed in this region, pushing the wall before it. Some of the reasons for this position were as follows:

The muscular elements of the sac were directly continuous with the uterine muscle. He did not believe that such a muscular sac could develop adventitiously in the abdominal cavity. He had seen cases of abdominal pregnancy in which the sac contained no muscular fibers. The head presentation deep down in the pelvic cavity, in the direction of the resultant of the forces developed by uterine contractions, supported his view of the case.

It is not necessary for the production of mural pregnancy that the tubes be involved. He thought there was much in the remarks of Dr. Nelson and Dr. Dudley. In cases in which there was sufficient contractility it was best to remove the placenta. Even under these circumstances it was not absolutely necessary. There was no danger in allowing the placenta to remain.

Finally, he was very positively of the belief that the two cases referred to in his paper, were examples of mural pregnancy. The peritoneum was a boundary line between mural and abdominal or peritoneal pregnancies.

Dr. Sawyer asked the question : Is the peritoneum a boundary line of importance in the macroscopical or microscopical differential diagnosis between abdominal and mural pregnancy?

Dr. Jaggard, in reply, said that the peritoneum was no barrier. What was the peritoneum? Dr. Etheridge, in an article on "Chronic Adhesive Perimetritis," published in a recent number of the CHICAGO MEDICAL JOURNAL AND EXAMINER, had ably sketched the anatomy of the membrane. It was developed out of connective tissue, according to Reindfleisch and other distinguished anatomists. It offered absolutely no barrier to the attachment of the ovum to the posterior uterine wall and its development in this situation, with the derivation of muscular elements from the normal uterus.

On motion, Doctors Byford, Merriman and Jaggard were appointed a committee to select a competent pathological anatomist, who did not belong to the Society, to examine the specimen and report at the next regular meeting. It was specified in the resolution that the *pathologist should be amply paid for the labor*.

Dr. Etheridge then exhibited a placenta with calcareous deposits. The placenta was removed from the body of a woman pregnant for the first time, who had probably carried the fœtus

292 days. The calcareous deposit was probably the result of fatty metamorphosis of the upper layers of the *decidua serotina.*

Dr. Sawyer said the placenta was interesting but not uncommon. It had been erroneously believed that such *placentæ* are of syphilitic origin. He thought the connection with prolonged gestation was established.

Dr. Dudley referred to the calcareous deposit in the walls of the arteries supplying an ovarian cyst, which he had removed some years previously.

Dr. Jackson related the history of a case, in which he had removed a mass of calcium carbonate, situated in the recto-vaginal septum, one and one-half inches from the vulvo-vaginal orifice. There was no fatty metamorphosis in this case.

Dr. Earle thought there was an unreasonable tendency to ascribe such cases to the effects of syphilis. Hydatidiform degeneration of the chorionic villi and *hydrops amnii* received a similar erroneous etiology.

Dr. Etheridge said that the deposits were composed of phosphate and carbonate of calcium. These salts had an affinity for albumens, and fatty acids present in the cotyledons. Similar calcareous deposits were met with in fibroids, thrombi, encysted trichinæ and in the lithopædia of extra-uterine pregnancy.

Dr. W. H. Byford thought the connection between prolonged gestation and calcareous deposits in the placenta was established. He thought Dr. Etheridge would find on microscopical examination that the changes had occurred exclusively within the vessel walls.

The Society then adjourned to meet on the third Friday evening in January, at the residence of Dr. E. C. Dudley, No. 2317 Indiana avenue, at 8 o'clock.

The business of next meeting will consist of

1. Report of the pathologist, Dr. Christian Fenger, on Dr. Byford's specimens.

2. Exhibition of specimens from a double ovariotomy, by Dr. E. C. Dudley.

3. Discussion of certain methods by which the second stage of labor may be rendered easier, by Dr. Henry F. Byford.

W. W. JAGGARD, M. D.,

Editor.

22nd Dec., 1884.

2330 Indiana avenue.

---

CHICAGO MEDICAL SOCIETY.

